# Real-time guava tree-part segmentation using fully convolutional network with channel and spatial attention

**DOI:** 10.3389/fpls.2022.991487

**Published:** 2022-09-13

**Authors:** Guichao Lin, Chenglin Wang, Yao Xu, Minglong Wang, Zhihao Zhang, Lixue Zhu

**Affiliations:** ^1^School of Mechanical and Electrical Engineering, Zhongkai University of Agriculture and Engineering, Guangzhou, China; ^2^Guangdong Laboratory for Lingnan Modern Agriculture, Guangzhou, China

**Keywords:** tree-part segmentation, MobileNetV3, attention mechanism, neural network, harvesting robot

## Abstract

It is imminent to develop intelligent harvesting robots to alleviate the burden of rising costs of manual picking. A key problem in robotic harvesting is how to recognize tree parts efficiently without losing accuracy, thus helping the robots plan collision-free paths. This study introduces a real-time tree-part segmentation network by improving fully convolutional network with channel and spatial attention. A lightweight backbone is first deployed to extract low-level and high-level features. These features may contain redundant information in their channel and spatial dimensions, so a channel and spatial attention module is proposed to enhance informative channels and spatial locations. On this basis, a feature aggregation module is investigated to fuse the low-level details and high-level semantics to improve segmentation accuracy. A tree-part dataset with 891 RGB images is collected, and each image is manually annotated in a per-pixel fashion. Experiment results show that when using MobileNetV3-Large as the backbone, the proposed network obtained an intersection-over-union (IoU) value of 63.33 and 66.25% for the branches and fruits, respectively, and required only 2.36 billion floating point operations per second (FLOPs); when using MobileNetV3-Small as the backbone, the network achieved an IoU value of 60.62 and 61.05% for the branches and fruits, respectively, at a speed of 1.18 billion FLOPs. Such results demonstrate that the proposed network can segment the tree-parts efficiently without loss of accuracy, and thus can be applied to the harvesting robots to plan collision-free paths.

## Introduction

Fruit harvesting is time-sensitive and labor-intensive, making manual picking expensive. In order to reduce the cost burden of manual picking, it is of great significance to develop intelligent harvesting robots. In structured environments, fruit trees are often planted in a V shape (Chen et al., 2021) or plane shape (Zhang et al., 2018), and fruit detection and localization are key problems facing the robots, which have been well-addressed. However, in unstructured environments, the fruit trees have complex three-dimensional structures, and therefore a major problem facing the robots is how to recognize tree parts (including fruits, branches, and backgrounds) for the robots to plan collision-free paths (Lin et al., 2021a). Due to the complex shape and uneven thickness of the branches, the tree parts are difficult to identify (Barth et al., 2018; Lin et al., 2021b). Guava is a fruit widely grown in Guangdong Province, China. In this study, a real-time and accurate guava tree-part segmentation method is investigated to enable the guava-harvesting robots to work in unstructured environments.

Tree-part segmentation can be accomplished by traditional image analysis methods, requiring manual design of classifiers *via* feature engineering (Amatya et al., 2016; Ji et al., 2016). Such methods are usually limited to specific environments and fruit trees. Currently state-of-the-art tree-part segmentation are dominated by fully convolutional networks (FCN). Our previous study used a VGG16-based FCN to segment guava branches with an intersection-over-union (IoU) of 47.3% and an average running time of 0.165 s (Lin et al., 2019). Furthermore, we employed Mask R-CNN to detect and segment guava branches simultaneously, and obtained 51.8% F1 score at a speed of 0.159 s per image (Lin et al., 2021b). Unfortunately, slender branches were found difficult to recognize. Li et al. deployed DeepLabV3 with Xception65 as the backbone to recognize litchi branches and fruits, and accomplished a mean IoU (mIoU) of 78.46% at a speed of 0.6 s (Li et al., 2020). Majeed et al. (2020) used a VGG16-based SegNet to segment tree trunk, branch and trellis wire, and achieved a boundary-F1 score of 0.93, 0.89, and 0.91, respectively. Zhang et al. employed DeepLabV3+ with a lightweight backbone ResNet18 to identify apple tree trunks and branches. The IoUs for trunks and branches were 63 and 40%, respectively, and the average running time was 0.35 s per image (Zhang et al., 2021). Chen et al. (2021) applied a ResNet50-based DeepLabV3, a ResNet34-based U-Net and Pix2Pix to segment occluded branches, respectively, and found that DeepLabV3 outperformed the other models in terms of mIoU, binary accuracy and boundary F1 score. Boogaard et al. (2021) segmented cucumber plants into eight parts by using a point cloud segmentation network PointNet++ and obtained 95% mIoU. Wan et al. developed an improved YOLOV4 to detect branch segments, applied a thresholding segmentation method to remove background, and used a polynomial fit to reconstruct the branches. The detection F1 score was 90%, and the running speed was 22.7 frames per second (FPS) (Wan et al., 2022). Because manually annotating a large empirical dataset is time-consuming and costly, Barth et al. trained DeepLabV2 with VGG16 as the backbone on a large synthetic dataset and then fine-tuned DeepLabV2 on a small empirical dataset. The final network categorized pepper plants into seven different parts with a mIoU of 40% (Barth et al., 2019). Furthermore, Barth et al. (2020) deployed a cycle generative adversarial network to generate realistic synthetic images to train DeepLabV2 and obtained 52% mIoU. Although the approaches mentioned above produce encouraging results, they are typically computationally inefficient since they employ very deep backbones to encode both low-level and high-level features. How to strike a balance between real-time performance and accuracy is a key problem that needs to be solved.

Recently, some efforts have been made to develop real-time segmentation networks. These efforts can be roughly divided into two categories. The first category uses existing lightweight backbones to reduce computation. Howard et al. (2019) developed a shallow segmentation head and appended it to the top of MobileNetV3, and achieved a mIoU of 72% with only 1.98 million multiply-accumulate operations on Cityscapes dataset. Hu et al. proposed a fast spatial attention module to enhance the features encoded by ResNet34, used a simple decoder to merge the features, and achieved 75.5 mIoU at 58 FPS on the Cityscapes dataset (Hu P. et al., 2020). Another category uses customized lightweight backbones to speed up the network inference. Yu et al. proposed a novel network termed BiSeNetV2, which uses a semantic branch with narrow channels and deep layers to generate high-level semantics, applies a detail branch with wide channels and shallow layers to obtain low-level details, and combines these features to predict a segment map. It achieves 72.6% mIoU on the Cityscapes dataset with a speed of 156 PFS (Yu et al., 2021). Gao (2021) proposed a fast backbone that consists of many dilated block structures and used a shallow decoder to output the segmentation. The network achieves 78.3 mIoU at 30FPS on the Cityscapes dataset. Overall, the first category is more attractive, because it utilizes exiting backbones to extract semantic features and hence allows us to focus on more important modules such as decoder.

The objective of this study is to develop a real-time and accurate tree-part segmentation network so that the robots can avoid the obstacles during harvesting. Specifically, a state-of-the-art lightweight backbone is deployed to capture the low-level and high-level features. And then, an attention module is proposed to enhance informative channels and locations in the above features. Subsequently, these features are fused together by a feature aggregation module. The final feature is processed by a segmentation head to output a segment map. A comprehensive experiment is performed to evaluate the proposed tree-part segmentation network.

The contribution of the study is listed as follows:

(1) A tree-part dataset containing 891 RGB images is provided, where each image is annotated on a per-pixel level manually.
(2) A real-time tree-part segmentation is proposed by improving an FCN with channel and spatial attention.
(3) The developed network achieves impressive results. Specifically, when using MobileNetV3-Large as the backbone, the network achieves an IoU of 63.33, 66.25, and 93.12% for the branches, fruits and background, respectively, at a speed of 36 FPS.

## Materials and methods

In this section, the data used for this research, including data acquisition, split and annotation, is presented in section 2.1. The developed tree-part segmentation network is introduced in section 2.2. Section 2.3 explains the evaluation criteria used to measure the performance of the developed network.

### Data

#### Data acquisition

**FIGURE 1:**
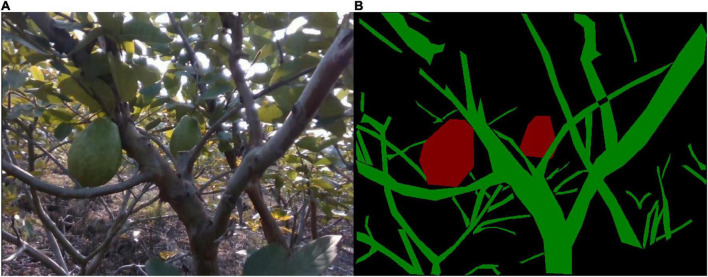
Image example. **(A)** A guava tree. **(B)** Different parts of the guava tree, where the red, green, and black regions represent the fruit, branch, and background, respectively.

The data acquisition site is located in a commercial guava orchard on Haiou Island, Guangzhou, China. The guava species is carmine. There is 3.1 m between two neighboring rows and 2.5 m between two neighboring trees in each row. A low-cost depth camera RealSense D435i is used to capture images, which can simultaneously generate RGB and depth images. This study only uses RGB images, which have a resolution of 480 pixels by 640 pixels. The images were taken on September 24, 2021 between 12:00 and 16:00, just in time for the guava harvest. The day was sunny with a temperature range of 30–34°C. During image acquisition, the camera was held by hand and moved along the path between two rows. The distance between camera and guava tree was about 0.6 m. A total of 41,787 images were acquired. Because adjacent images look similar and may have little effect on network training, a subset of the images were sampled uniformly which comprises 891 images. Figure 1A shows a captured image.

#### Data split

These 891 RGB images were divided into a test and training set. The test set contains the first 30% of the images, and the training set contains the last 70% of the images. This partitioning approach keeps the data sets independent and therefore better examines the generalization performance of the network.

#### Data annotation

Because branches and fruits will prevent the robots from getting close to the targets, they should be annotated to enable the network to recognize them. Each pixel on the images in the training and test sets was annotated as a branch, fruit, or background class using the open-source annotation program LabelMe (Russell et al., 2008). A visual example is shown in Figure 1B. It is worth noting that per-pixel label annotation is very time-consuming, and we spent almost 2 months to accomplish the annotation task.

### Tree-part segmentation network

This section illustrates the proposed tree-part segmentation network in detail. An efficient network backbone for capturing low-level and high-level features is introduced in Section 2.2.1. The proposed channel and spatial attention module for boosting meaningful features is elaborated in Section 2.2.2. Section 2.2.3 describes the multi-level feature aggregation module for fusing low-level details and high-level semantics. Section 2.2.4 introduces the segmentation head, and Section 2.2.5 presents the network architecture.

#### Backbone

To realize real-time segmentation and thus enable the harvesting robots to work efficiently, an efficient neural network MobileNetV3 (Howard et al., 2019) is employed as the segmentation network backbone. MobileNetV3 builds on the latest techniques such as depth-wise separable convolution, inverted bottleneck (Sandler et al., 2018) and squeeze-excitation network (Hu J. et al., 2020), and has been widely deployed in mobile applications. There are many layers outputting feature maps of the same resolution, and these layers are considered to be at the same stage. MobileNetV3 has five stages. Let {*C*_2_, *C*_3_, *C*_4_, *C*_5_} denote the outputs of the last layer of stage 2, stage 3, stage 4, and stage 5. Typically, the output of shallow stage such as *C*_2_ contains low-level information but with limited semantics, while that of deep stage such as *C*_5_ contains high-level semantics but with low resolution. These low-level details and high-level semantics can be combined to achieve high accuracy segmentation (Yu et al., 2021). Therefore, they are utilized in this study.

Because MobileNetV3 is primitively designed to output 1,000 classes for ImageNet (Russakovsky et al., 2015), the last few layers have many channels, which may be redundant for our task. In this study, the last layer in stage 5 is directly excluded. We discover that this modification can improve the segmentation accuracy and speed. Additionally, it is a common practice to place atrous convolution in the last few stages of the backbone to generate dense feature maps, which can effectively increase the segmentation accuracy (Chen et al., 2018; Howard et al., 2019). However, when we developed the network model in this paper, we found that the atrous convolution harmed the performance of our network. Hence, we do not use it in the backbone.

Global context information can reduce the probability of misclassification. Pyramid pooling module (PPM) (Zhao et al., 2016) is a practical technique to generate global context information, which uses four different scales of global average pooling layers to enlarge the network receptive fields, up-samples the resulting feature maps so that they have the same size as the original feature map by bilinear interpolation, and then concatenates them as the final global context information. PPM is attached at the top of MobileNetV3.

#### Channel and spatial attention module

Formally, {*C*_2_, *C*_3_, *C*_4_, *C*_5_} encode different levels of channel and spatial information. Not every channel offers useful information. Channel attention mechanism (Roy et al., 2018; Woo, 2018; Hu J. et al., 2020) can be used to recalibrate these feature maps to focus on useful channels, thereby increasing the representation power. Note that the squeeze and excitation attention block of MobileNetV3 serves to refine some intermediate layers, whereas the channel attention mechanism here only serves to refine the output of the last layer of each stage. Besides, the pixel-wise spatial information is more important for semantic segmentation. Therefore, the feature maps can be further recalibrated along space using spatial attention mechanism, making them more informative spatially (Roy et al., 2018; Woo, 2018). To this effect, a channel and spatial attention module (CSAM) is proposed, which consists of a channel attention module and a spatial attention module. CSAM is detailed as follows.

The channel attention module is developed by the inspiration of Howard et al. (2019) to strengthen useful channels and weaken useless channels. Let **X** ∈ ℝ*^H × W × C^* denote a feature map, where *H* and *W* are the spatial height and width, and *C* is the number of channels. A global average pooling layer is first performed on ***X***, resulting a vector **u** ∈ ℝ^*C*^ with its *k^th^* element:


(1) $$u_{k}=\frac{1}{H\times W}\sum_{h=1}^{H}\sum_{w=1}^{W}\text{u}\left(h,w,k\right)$$

Vector **u** is then used to generate a gate vector ***g*** by employing a gating mechanism:


(2) $$\text{g}=\sigma \left({\text{W}}_{1}\text{u}\right)$$

where σ refers to the sigmoid function, ${\text{W}}_{1}\in {\mathbb{R}}^{\frac{C}{r}\times C}$ is a learnable tensor, and *r* is a reduction ratio using for limiting model complexity. Gate vector ***g*** measures the usefulness of the channels, which is used to recalibrate ***X***:


(3) $${\text{X}}_{c}=\text{g}⊗\delta \left({\text{W}}_{2}*\text{X}\right)$$

where ⊗ denotes the channel-wise multiplication, δ is the ReLu function, * refers to convolution, ${\text{W}}_{2}\in {\mathbb{R}}^{1\times 1\times C\times \frac{C}{r}}$ denotes the filter kernel, and ${\text{X}}_{c}\in {\mathbb{R}}^{H\times W\times \frac{C}{r}}$ is the projection of ***X***. Equation 3 not only depicts the interdependencies between the channels of X, but also highlights the useful channels while downplaying the useless ones.

In order to fully exploit the spatial information of the feature map, the spatial attention module developed by Roy et al. (2018) is deployed. Specifically, a gate map **G** ∈ ℝ*^H × W^* is first generated *via* squeezing the feature map along its channel dimension and employing a sigmoid function:


(4) $$\text{G}=\sigma \left({\text{W}}_{3}*{\text{X}}_{c}\right)$$

where ${\text{W}}_{3}\in {\mathbb{R}}^{1\times 1\times \frac{C}{r}\times 1}$ is the filter kernel. Then gate map ***G*** is used to rescale the feature map:


(5) $${\text{X}}_{s}=\text{G}⊗{\text{X}}_{c}$$

where ⊗ denotes the element-wise multiplication. Equation 5 makes the network focus on important spatial locations and ignore useless ones.

**FIGURE 2:**
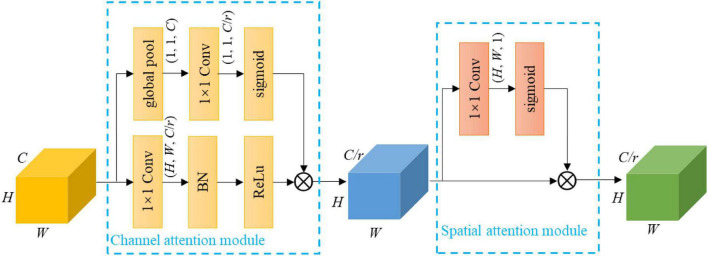
Design details of CSAM. Note that *Conv* is convolutional operation, and BN is batch normalization; 1 × 1 represents the kernel size, *H* × *W* × *C* and *H* × *W* × *C*/*r* denote the tensor shape (height, width, and depth); the first ⊗ refers to channel-wise multiplication, and the second ⊗ is element-wise multiplication.

The architecture of CSAM is illustrated in Figure 2. CSAM is appended on *C*_2_, *C*_3_, *C*_4_ and the output of PPM, and the corresponding reduction ratios are set to {1, 1, 2, 4} for MobileNetV3-Large and {1, 1, 1, 2} for MobileNetV3-Small. The resulting feature maps are denoted as {*G*_2_, *G*_3_, *G*_4_, *G*_5_}. It is worth noting that CASM is attached to PPM and not *C*_5_ simply because PPM itself contains *C*_5_. The work (Roy et al., 2018) also proposes a similar attention module. CSAM differs in introducing a reduction ratio to reduce the module complexity, and information goes through the two modules in an orderly manner, which progressively filters out useless information.

Let us consider an input feature map of *C* channels. The channel attention module introduces $\frac{2C^{2}}{r}$ new weights, while the spatial attention module introduces $\frac{C}{r}$ weights. So, a CASM brings a total of $\frac{2C^{2}+C}{r}$ parameters. Because the feature maps of MobileNetV3 have relatively few channels, these extra parameters only add a small amount of computation to the backbone.

#### Feature aggregation module

**FIGURE 3:**
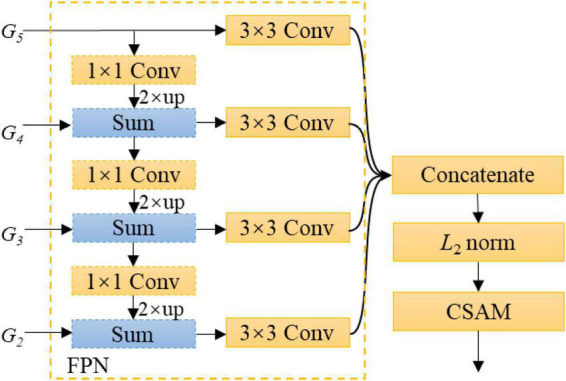
Design details of FAM. Note that *up* refers to up-sampling by bilinear interpolation.

Typically, thin branches are harder to segment than thick branches, because detailed information is easily lost when the output stride is increased. This problem can be alleviated by fusing feature maps from different layers, such as {*G*_2_, *G*_3_, *G*_4_, *G*_5_}. A simple variant of feature pyramid network (FPN) (Lin et al., 2016) is used to gradually up-samples and merges the feature maps from deepest feature maps to shallow ones. As shown in Figure 3, our FPN variant first appends a 1 × 1 convolutional layer on the coarsest feature map *G*_5_ to reduce its channel dimension, up-samples *G*_5_ by a factor of 2, and then merges *G*_5_ with its corresponding bottom-up map *G*_4_ by element-wise addition. This process is repeated until the finest feature map is generated. A 3 × 3 convolutional layer is appended on each merged feature map to generate the final feature map with a fixed output dimension of 48. Here, batch normalization and ReLu are adopted after each convolution, which are omitted for simplifying notations. On this basis, these feature maps are concatenated. Because lower-level feature maps may have large values than higher-level ones, which probably destabilizes network training, the concatenated features should be normalized carefully. To this effect, a *L*_2_ normalization layer (Liu et al., 2015) is performed on the concatenated features. Specifically, let **X** = (**x**_1_, …, **x**_*C*_) be the concatenated features, and *C* is the number of channels. ***X*** is normalized with the following equation:


(6) $${\text{x}}_{c}=\gamma_{c}\frac{{\text{x}}_{c}}{{||{\text{x}}_{c}||}_{2}}$$

where ||⋅||_2_ means the *L*_2_ norm; *c* = 1, …, *C*; and γ_*c*_ is a learnable scaling parameter, which can avoid the resulting features being too small and hence promotes network learning. In experiments, the initial value of γ_*c*_ is set to 1. Subsequently, a CSAM with reduction ratio of *K* is attached after the *L*_2_ normalization layer to further refine the feature map, where *K* refers to the number of feature maps fused. Figure 3 shows the architecture of the proposed FAM.

#### Segmentation head

**FIGURE 4:**
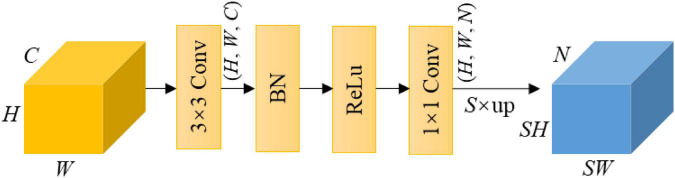
Illustration of the segmentation head. Note that *S* is the scale ratio of up-sampling, and *N* is the number of classes.

The segmentation head is used to output a segment map of the same size as the input RGB image, which is *N*-channeled with *N* being the number of classes. In this study, *N* equals to 3. Figure 4 shows the segmentation head, which consists of a 3 × 3 convolution layer, a batch normalization layer, a ReLU activation, a 1 × 1 convolution layer and an up-sampling operation *via* bilinear interpolation.

#### Network architecture

**FIGURE 5:**
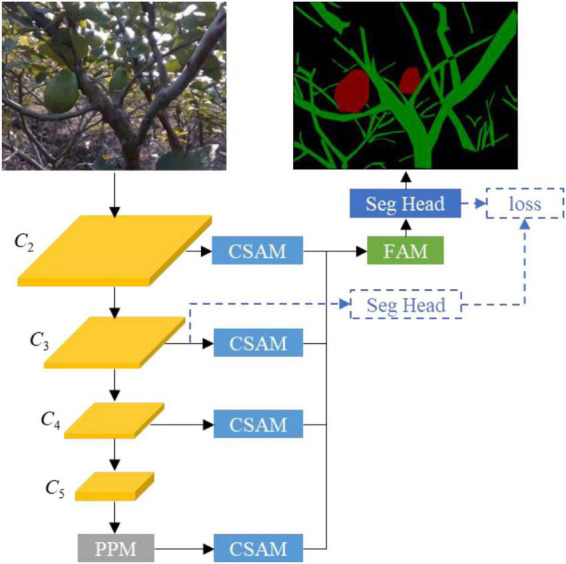
Overview of the tree-part segmentation network, where three-dimensional blocks represent feature maps and two-dimensional blocks refer to convolutional modules.

The overall architecture is shown in Figure 5. MobileNetV3 forms the backbone network with PPM attached on the top to capture global contextual information. Feature maps from the last layers of stage 2, stage 3, stage 4, and PPM are refined by CSAM and then used as input to FAM to produce a feature map containing low-level details and high-level semantics. The output of FAM is processed by the segmentation head to make the final semantic segmentation.

The tree-part segmentation network is trained in an end-to-end manner to minimize a cross-entropy loss defined on the output of the segmentation head. To stabilize network training, an auxiliary segmentation head is inserted after the output of stage 3, and an auxiliary cross-entropy loss with weight 0.4 is added to the final loss (Zhao et al., 2016), as shown in Figure 5. This auxiliary segmentation head is only used in the training phase and removed in the inference phase. Furthermore, a *L*_2_ regularization with weight 5e^–4^ on the parameters of the network except the backbone are added to the final loss to alleviate network over-fitting. Note that because this study uses a pre-trained MobileNetV3 on ImageNet as the backbone, we do not place the *L*_2_ regularization on the parameters of the backbone.

### Segmentation evaluation

To evaluate the accuracy performance of the tree-part segmentation network, three commonly used metrics are used: IoU, mIoU, and pixel accuracy (PA). For the sake of explanation, let *N* denotes the total number of classes, and *p*_*ij*_ denote the number of pixels that belong to class *i* but are predicted to be class *j*. Obviously, *p*_*ii*_, *p*_*ij*_ and *p*_*ji*_ represent the number of true positives, false negatives, and false positives, respectively. IoU is the ratio between the intersection and union of the ground true and predicted segmentation, and can be calculated by dividing true positives by the sum of false positives, false negatives and true positives. For class *i*, its IoU is computed as follows:


(7) $$IoU_{i}=\frac{p_{ii}}{\sum_{j=0}^{N-1}p_{ij}+\sum_{j=0}^{N-1}p_{ji}-p_{ii}}$$

mIoU is an improved IoU which computes the IoU value for each class and then averages them:


(8) $$mIoU=\frac{1}{N}\sum_{i=0}^{N-1}\frac{p_{ii}}{\sum_{j=0}^{N-1}p_{ij}+\sum_{j=0}^{N-1}p_{ji}-p_{ii}}$$

PA measures the network recall ability. It calculates a ratio between the amount of true positives and the total number of pixels:


(9) $$PA=\frac{\sum_{i=0}^{N-1}p_{ii}}{\sum_{i=0}^{N-1}\sum_{j=0}^{N-1}p_{ij}}$$

To measure the real-time performance of the developed network, three metrics are utilized: floating point operations per second (FLOPs), FPS, and number of parameters. Note that FPS is determined by counting how much RGB images can be processed per second in the inference phase.

## Experimental setup

### Implementation details

The developed network is programmed in Pytorch and runs on a computer with Windows 10 system, 32 GB RAM, Intel i9-11900K CPU, and NVIDIA GeForce RTX 3080 GPU. The backbone is pre-trained on ImageNet, and other parameters are initialized using the default initialization method in Pytorch. Standard Adam is used to minimize the loss function, and “cosine” learning scheduler (Loshchilov and Hutter, 2016) is used to adjust learning rate, where initial learning rate is set to 1e^–4^. The network is trained on the train set, and 150 training epochs are used with a mini-batch size of 12. To avoid network over-fitting, the following data augmentation methods are implemented during training: horizontal flipping, vertical flipping, random rotation within the range of [−45°, 45°], random scale within the rage of [0.8, 1.2], and randomly changing the hue, saturation and value of the input image.

### Ablation study

This section performs the ablation study to validate the effectiveness of each module in our network. In the following experiments, MobileNetV3-Large is used as the backbone, and the segmentation models are trained on our training set and evaluated on our test set. The ablation study is detailed as follows:

(1) Ablation for backbone. Placing atrous convolution in the last stage of the backbone can preserve the details, which has been widely utilized in semantic segmentation (Chen et al., 2018; Howard et al., 2019). However, it is unclear whether atrous convolution can improve the segmentation accuracy of our network. In addition, whether removing the last layer of stage 5 of the backbone network will improve efficiency and accuracy. Experiments are conducted to answer these questions.
(2) Ablation for feature aggregation. High-level features contain semantic information but with limited details, while low-level features contain detailed information but with limited semantics. Fusing these features can improve segmentation accuracy. However, it is unclear which low-level and high-level features should be fused. We re-implement the network with different combinations of the low-level and high-level features, and find the best combination through experiments.
(3) Ablation for auxiliary segmentation head. Auxiliary segmentation head has been widely used in semantic segmentation (Zhao et al., 2016; Yu et al., 2021). We insert the auxiliary segmentation head to different stages of the backbone in the training phase and reveal which position is most important.

### Comparison with existing methods

To evaluate the accuracy and real-time performance of the developed network, a comparison experiment is performed. MobileNetV3-Large and MobileNetV3-Small are used as the backbone of our network. Four state-of-the-art networks are used for comparisons: DeepLabV3 (Chen et al., 2017), DeepLabV3+ (Chen et al., 2018), LR-ASPP (Howard et al., 2019), and FANet (Hu P. et al., 2020). For the sake of comparison, DeepLabV3, DeepLabV3+ and LR-ASPP use MobileNetV3-Large as the backbone, and apply the atrous convolution to the last block of MobileNetV3-Large to generate denser feature maps. FANet uses ResNet18 as the backbone as suggested by Hu P. et al. (2020). All of the comparison networks are implemented in Pytorch and trained according to the strategy described in section 3.1. Our network and the comparison networks are evaluated on the test set, and quantitative results including IoU, mIoU, PA, FPS, and FLOPs are reported and discussed.

## Results and discussion

### Ablation study

Table 1 lists the results of different configurations of the backbone. As shown in the table, we observed that (1) when not employing the atrous convolution in the last block of the backbone to extract dense features, the mIoU and PA slightly improved by 0.20 and 0.19%, respectively, while being faster (row 1 vs. row 3), (2) removing the last layer in stage 5 of the backbone did not decrease the IoU and PA while being slightly faster (row 1 vs. row2, row 3 vs. row 4), and (3) when not employing the atrous convolution and removing the last layer in stage 5, the network obtained similar accuracies while being significant faster than its variants (row 4 vs. row 1, 2, and 3). These results indicate that the atrous convolution was not necessary for our task, and the MobileNetV3 backbone contained redundant layers which should be excluded.

**TABLE 1:** Ablations on the backbone and feature aggregation module.

Row	AC	R	NF	IoU (%)			mIoU (%)	PA (%)	FPS	#Params	FLOPs
				Branch	Fruit	Background					
1	✓	x	4	63.37	66.67	93.05	74.03	93.76	32.84	6.9M	3.48B
2	✓	✓	4	62.51	67.05	93.18	74.25	93.87	33.85	5.7M	3.08B
3	x	x	4	63.40	66.03	93.26	74.23	93.95	33.80	6.9M	2.44B
4	x	✓	4	63.33	66.25	93.12	74.23	93.84	36.00	5.7M	2.36B
5	x	✓	3	58.72	63.14	92.20	71.35	92.96	34.67	5.7M	1.66B
6	x	✓	2	49.74	61.16	90.72	67.21	91.49	34.36	5.7M	1.46B

AC, Apply atrous convolution in the last block of the backbone; R, Remove the last layer in stage 5 of the backbone; NF, Number of feature maps fused in FAM. When NF = 4, {*G*_2_, *G*_3_, *G*_4_, *G*_5_} are fused. When NF = 3, {*G*_3_, *G*_4_, *G*_5_} are fused. When NF = 2, {*G*_4_, *G*_5_} are fused. M and B represent million and billion, respectively.

Aggregating different levels of features has varying effects on the network performance, as shown in Table 1. Fusing {*G*_2_, *G*_3_, *G*_4_, *G*_5_} performed better than fusing {*G*_3_, *G*_4_, *G*_5_} and {*G*_4_, *G*_5_} by 2.88 and 7.02%, respectively, in terms of mIoU, and only required a few more computation. This illustrates that the network performance could benefit from fusing as many features as possible. In this study, we fused {*G*_2_, *G*_3_, *G*_4_, *G*_5_} to improve the network accuracy.

Table 2 shows the effect of different positions to place the auxiliary segmentation head. As can be seen, inserting the auxiliary segmentation head into the output of stage 3 outperformed that of stage 2, stage 4 and stage 5 by 0.65, 1.12, and 1.47%, respectively, in terms of mIoU, and slightly underperformed that of stage 4 and stage 5 by 0.17 and 0.08%, respectively, in terms of PA. Therefore, we chose to attach the auxiliary segmentation head to the output of stage 3.

**TABLE 2:** Ablations on the auxiliary segmentation head, which is inserted after the output of different stages in the backbone.

Stage	IoU (%)			mIoU (%)	PA (%)
	Branch	Fruit	Background		
2	62.45	65.32	92.95	73.58	93.68
3	63.33	66.25	93.12	74.23	93.84
4	64.04	61.96	93.33	73.11	94.02
5	63.07	61.98	93.23	72.76	93.92

### Comparison with existing methods

Table 3 lists the accuracy and real-time performance of the proposed network and comparison methods. Overall, our network with MobileNetV3-Large as the backbone outperformed LR-ASPP, DeepLabV3, DeepLabV3+, and FANet in terms of the accuracy metrics, which validated the effectiveness of the proposed modules. Furthermore, our network performed faster than DeepLabV3, DeepLabV3+ and FANet in terms of FLOPs, likely because DeepLabV3 and DeepLabV3+ applied a very time-consuming atrous spatial pyramid pooling module to encode context information, and FANet used a relatively large backbone. Surprisingly, there was little difference in FPS between our network and the comparison networks, probably because the depth-wise convolution in MobileNets and the multi-branch design in ResNet increased the memory access cost, affecting the inference speed (Ding et al., 2021). Conclusively, the proposed network with MobileNetV3-Large as the backbone was more accurate than the comparison methods while being fast.

**TABLE 3:** Accuracy and real-time performance of the proposed network and comparison methods on test set.

Methods	Backbone	IoU (%)			mIoU (%)	PA (%)	FPS	#Params	FLOPs
		Branch	Fruit	Background					
Ours	MobileNetV3-Large	63.33	66.25	93.12	74.23	93.84	36.00	5.7M	2.36B
Ours	MobileNetV3-Small	60.62	61.05	92.82	71.50	93.52	37.91	2.7M	1.18B
LR-ASPP	MobileNetV3-Large	60.05	58.60	92.85	70.50	93.52	36.67	5.7M	2.37B
DeepLabV3	MobileNetV3-Large	56.34	58.82	92.14	69.11	92.85	35.78	13.5M	11.58B
DeepLabV3+	MobileNetV3-Large	62.59	61.05	93.36	72.33	94.00	31.52	14.2M	35.73B
FANet	ResNet18	54.71	57.57	92.25	68.17	92.97	36.65	13.8M	6.93B

Additionally, our network with MobileNetV3-Small as the backbone had slightly lower accuracy than DeepLabV3+, but higher accuracy than LR-ASPP, DeepLabV3, and FANet. Moreover, this network achieved the best real-time performance. In other words, when MobileNetV3-Small was used as the backbone, the proposed network was the fastest among the comparison networks, but somewhat less accurate.

Our network achieved a large IoU value for the background, probably because the background dominated the images, making the network pay more attention to the background. This problem can be alleviated by reshaping the loss function by down-weighting the background and up-weighting other objects (Ronneberger et al., 2015). Besides, the IoU value of the branch class was lower than that of the fruit class. A possible reason was that some branches were very thin and hence their detailed information was easy to be lost, making them hard to segment. Although we have fused multi-layer features to solve such a problem, MobileNetV3 was too lightweight to provide enough features. Future work will consider adding a detail branch (Yu et al., 2021) to the backbone to extract detailed information.

**FIGURE 6:**
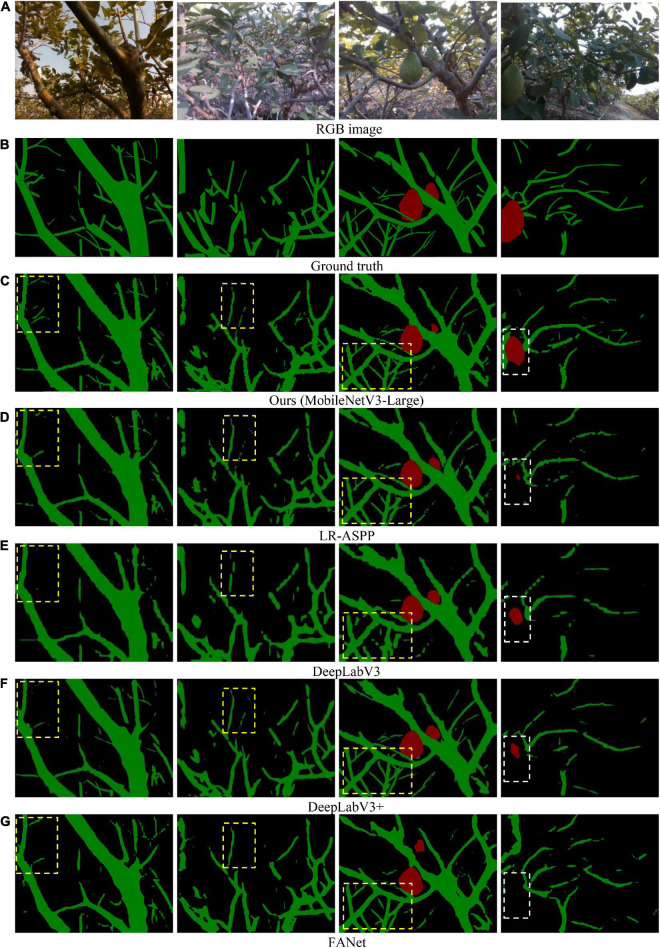
Visual examples illustrating results of our network and comparison networks. **(A)** RGB image. **(B)** Ground truth. **(C)** Ours (MobileNetV3-Large). **(D)** LR-ASPP. **(E)** DeepLabV3. **(F)** DeepLabV3+. **(G)** FANet.

Some qualitative results were shown in Figure 6. Visually, our network was more accurate in tree-part segmentation. Specifically, the developed network could capture the details of most thin branches, whereas the comparison networks struggled to segment the thin branches, as shown in the yellow boxes in columns 1–3 of Figure 6. Besides, our network outperformed the comparison networks in the recognition ability of fruits, as shown in the while boxes in column 4 of Figure 6. The results validate the effectiveness of the developed attention module and feature aggregation module. Although most of the branches were identified, some thin branches seemed to be difficult to identify. In robotic harvesting, the thin branches might clog the end effector, causing shear failure. Therefore, future work will focus on improving the segmentation accuracy of thin branches. A relevant video can be found at: https://www.bilibili.com/video/BV1nS4y147wa/?vd_source=d082953b9cfe065d2d003486f259e84f.

## Conclusion

This study aimed to develop a tree-part segmentation network that can segment fruits and branches efficiently and accurately for harvesting robots to avoid obstacles. Experimental results validated that the proposed network can accomplish the research objective. Some specific conclusions drawn from the study were given as follows:

(1) A tree-part dataset was collected. The dataset consists of 891 RGB images captured in the fields. Each image is manually annotated in a per-pixel fashion, which took us almost 2 months to label. To the best of our knowledge, this is the first tree-part dataset used to help harvesting robots avoid obstacles.
(2) A tree-part segmentation network was developed, which consists of four components: a lightweight backbone, CASM, FAM, and segmentation head. Here, CASM was used to enhance informative channels and locations in the feature maps, and FAM was designed to fuse multi-layer feature maps to improve the segmentation accuracy. Experiments on the test set shows that when using MobileNetV3-Large as the backbone, the network achieved an IoU of 63.33, 66.25, and 93.12% for the branches, fruits and background, respectively, at a speed of 2.36 billion FLOPs. These performance values validates that the network could segment tree parts efficiently and quite accurately. However, the IoU value of the branch class was the lowest, probably because the max-pooling operations in the backbone lost the detailed information of the thin branches, thus making the thin branches difficult to segment.

The proposed network could be transferred to segment other fruits by fine-tuning on new datasets. Future research will add two more classes (soft branch and hard branch) to the current dataset to allow harvesting robots to push away soft branches and avoid hard ones for better fruit picking. Furthermore, future work will attempt to add a detailed path in the backbone to preserve the detailed information of the input image, thus improving the accuracy.

## Data availability statement

The original contributions presented in this study are included in the article/supplementary material, further inquiries can be directed to the corresponding authors.

## Author contributions

GL: methodology, investigation, and writing—original draft. CW: investigation, methodology, and writing—review and editing. YX and MW: writing—review and editing. ZZ: conceptualization and data curation. LZ: methodology and supervision. All authors contributed to the article and approved the submitted version.
